# Molecular Characteristics of Jujube Yellow Mottle-Associated Virus Infecting Jujube (*Ziziphus jujuba* Mill.) Grown at Aksu in Xinjiang of China

**DOI:** 10.3390/v13010025

**Published:** 2020-12-25

**Authors:** Jiashu Guo, Yanxiang Wang, Guoping Wang, Jian Hong, Zuokun Yang, Jianyu Bai, Ni Hong

**Affiliations:** 1 Key Lab of Plant Pathology of Hubei Province, College of Plant Science and Technology, Huazhong Agricultural University, Wuhan 430070, China; guojiashu94@163.com (J.G.); wangyanxiang@webmail.hzau.edu.cn (Y.W.); gpwang@mail.hzau.edu.cn (G.W.); 13297974203@163.com (Z.Y.); 2 Key Laboratory of Horticultural Crop (Fruit Trees) Biology and Germplasm Creation of the Ministry of Agriculture, College of Horticulture and Forestry Sciences, Huazhong Agricultural University, Wuhan 430070, China; 3 Analysis Center of Agrobiology and Environmental Sciences, Zhejiang University, Hangzhou 317502, China; jhong@zju.edu.cn; 4 Laboratory of Fruit Trees Disease, Institute of Economic Forestry, Xinjiang Academy of Forestry Sciences, Urumqi 830063, China

**Keywords:** jujube, high-throughput sequencing, *Emaravirus*, genetic diversity

## Abstract

Chinese jujube (*Ziziphus jujuba* Mill.) is a native fruit crop in China. Leaf mottle and dapple fruit disease is prevalent in cultivated jujube plants grown at Aksu in Xinjiang Uygur Autonomous Region of China. Jujube yellow mottle-associated virus (JYMaV), a tentative member in the genus *Emaravirus*, was recently identified from mottle-diseased jujube plants grown in Liaoning Province in China, but its incidence and genetic diversity in China is unknown. In this study, the genome sequences of three JYMaV isolates from two jujube cultivars and one jujube variant were determined by high-throughput sequencing (HTS) for small RNA and rRNA-depleted RNA coupled with RT-PCR assays. Comparison of these sequences together with sequences of the viral RNA segments derived by primer set 3C/5H-based RT-PCR revealed that genetic diversity was present in the virus populations and high sequence variation occurred at the non-translational regions of each of the viral genomic segments. Field investigation confirmed the close association of the virus with leaf mottle symptoms of jujube plants. Furthermore, this study revealed that P5 encoded in the viral RNA5 displayed a nuclear localization feature differing from the plasmodesma (PD) subcellular localization of the virus movement protein (P4), and the two proteins could interact with each other in the BiFC assays. Our study provides a snapshot of JYMaV genetic diversity in its natural hosts.

## 1. Introduction

Chinese jujube (*Ziziphus jujuba* Mill.), a fruit crop in the family *Rhamnaceae* with a cultivation history dating back over 7000 years, is native to China [1,2], where it is widely cultivated [3]. Chinese jujube has become one of the most common and economically important fruit crop species in the Xinjiang Uygur Autonomous Region of China. For a long time, jujube witches broom caused by *Phytoplasma* sp. is considered as a major threat to jujube production in some jujube producing areas [4,5]. In recent years, a jujube mosaic disease has been observed in several jujube-growing regions in China. By using high throughput sequencing (HTS) combined with RT-PCR amplification, a tentative badnavirus named jujube mosaic-associated virus (JuMaV) and a novel emaravirus tentatively named jujube yellow mottle-associated virus (JYMaV) were respectively identified from mosaic-diseased jujube plants grown in Beijing City and Liaoning Province in China [6,7]. However, the association of JYMaV with the jujube mosaic disease reminds for further investigation.

The genus *Emaravirus* belongs to the newly established family *Fimoviridae* in the order *Bunyavirales* [8,9]. The genomes of viruses in the genus *Emaravirus* are characterized by having 4–8 single-stranded negative-sense genomic RNA (-ssRNA) segments, each containing a single open reading frame (ORF) in the complementary strand and flanked by two untranslated regions. All genomic RNAs of emaraviruses are not capped or polyadenylated, but contain complementary 13-nt stretches at their 5′ and 3′ termini, which are highly conserved in emaraviruses [10]. Four core proteins consisting of RNA-dependent RNA polymerase (RdRp), glycoprotein (GP) precursor, nucleocapsid protein (NP), and movement protein (MP) are encoded in RNAs 1–4 and are essential for the replication, virion assembling and movement [8,10]. Except for Palo Verde witches broom virus (PVWBV), for which four genomic RNA segments have been reported, most emaraviruses possess additional genomic RNA segments encoding proteins with unknown function [8,11,12]. It has been reported that some emaraviruses are transmissible by vectoring of eriophyid mites (Acari: *Eriophyidae*) [10,13,14,15,16,17,18]. Then, eriophyid mites may play important role in the naturally spreading of emaraviruses among host plants.

Recently, leaf mosaic and fruit blotch disorder disease was found to be widely distributed in the major jujube growing areas at Aksu in Xinjiang Uygur Autonomous region of China and caught great attention for producers and scientists. Since the diseased trees showed systemic disease symptoms, highly like viral diseases of jujube recently reported in China [6,7], to understand the causal agents of the disease, high-throughput sequencing (HTS) was carried out for small RNA (sRNA) and RNA extracted from three leaf samples of diseased Chinese jujube plants. Our results revealed the presence of JYMaV in the diseased samples. Nowadays, JYMaV has been only identified from jujube trees suffering leaf mosaic disease in Liaoning Province in China and the genomic sequence of one JYMaV isolate SY is available in GenBank database [7]. The genetic variability of JYMaV is rarely known. In this study, for the first time, we reported the wide occurrence of JYMaV in jujube trees grown at Aksu in Xinjiang and the molecular diversity of the viral genomic RNAs.

## 2. Materials and Methods

### 2.1. Samples Used for Small RNA and RNA Sequencing

In 2017 and 2018, leaf samples were collected from three single plants of *Z. jujuba* cv. ‘Huizao’, *Z. jujuba* cv. ‘Junzao’ and *Z. jujuba* var. ‘spinosa’ (sample IDs: HZ, AKS-6 and AKS-15) grown at Aksu, Xinjiang Uygur Autonomous Region, China. These plants exhibited symptoms of semi-transparent chlorotic spots and blotches on young leaves and the diseased leaves were usually malformed. The cultivars ‘Huizao’ and ‘Junzao’ are widely grown at Aksu. Seedlings of *Z. jujuba* var. ‘spinosa’ are popular rootstocks of cultivated jujube plants. The sample HZ was used to generate a cDNA library of small RNAs (sRNAs) as reported previously [19]. After the identification of JYMaV in the sample HZ, the samples AKS-6 and AKS-15 were subjected to RNA-Seq analyses for further characterization of the viral genomic RNAs.

### 2.2. Small RNA and RNA Sequencing

The generations of a cDNA library of sRNAs from HZ and two cDNA libraries of rRNA-depleted RNAs from AKS-6 and AKS-15 were performed by Biomarker Biology Technology Ltd. Company (Beijing, China) as reported previously [19,20]. For RNA-Seq, ribosomal RNA (rRNA) in total RNA extracts was removed using an Epicentre Ribo-ZeroTM rRNA removal kit (Epicentre, Madison, WI, USA). The prepared rRNA-depleted RNA samples were used to construct cDNA libraries with a TruSeq RNA Sample Prep Kit v2 (Illumina, San Diego, CA, USA) and sequenced on an Illumina HiSeq XTen sequencing machine (Illumina) with a paired-end 150 bp setup (Biomarker Biology Technology Ltd. Company, Beijing, China).

The raw sRNA reads and RNA reads from the Illumina platform were trimmed of adaptor sequences and filtered for low-quality reads using FASTP version 1.5.6 to remove adapter sequences and reads with more than 5% Ns or with 20% base quality values (Q20) less than 20. For sRNA data, reads below 15 and above 30 nt were discarded. Then, the trimmed sRNA reads and RNA reads were de novo assembled into larger contigs using Velvet version 1.2.08 [20] with a k-mer of 15–17 and IDBA-UD version 1.1.1 [21] with k-mer values of 80, 90 and 110, respectively. Contigs were subsequently screened for sequence identities against the NCBI databases (http://www.ncbi.nlm.nih.gov/ accessed on and 10 September 2017 and 15 September 2019) using BlastX and BlastN programs.

### 2.3. Determination of Viral Genome

Total RNA was extracted from the sample HZ using a silica spin column-based RNA isolation method [22]. Reverse transcription was performed using a universal primer 3C targeting to the 3′ end sequence of emaraviruses [19]. The resulting cDNA was used as templates to generate the viral genome sequences. Basing on the feature that nucleotide sequences at both 5′ and 3′ termini of the genomic RNAs of emaraviruses are highly conserved and complementary reversely to each other, the primer set 5H/3C, which targets the terminal regions of emaraviruses RNAs [19], was used to amplify the genomic RNAs of the identified emaravirus except for its RNA1. For the amplification of the viral RNA1, cDNA was generated using random hexamer pd(N)6 primers (TaKaRa, Dalian, China). Six pairs of specific primers were designed according to the sequences of assembled contigs, which were mapped to genomic RNA1 of JYMaV. Meanwhile, a primer set AF/CR was used to amplify the sequence conserved in RdRp coding region of emaraviruses [19]. Finally, the terminal sequences of genomic RNA3 were determined with the RACE strategies using a commercial kit (TaKaRa, Dalian, China) according to the manufacturer’s instructions. The adjacent fragments were overlapped for at least 100 bp. The primers used for the amplification of the viral genomic RNA segments were listed in the Table 1.

RT-PCR solutions (25 μL) contained 2 μL of cDNA template, 2.5 μL of 10 × PCR buffer, 10 mM of dNTPs, 10 pM of each of forward and reverse primer and 1U of r*Taq* DNA polymerase (TaKaRa). RT-PCR conditions were like those reported previously [19], except that annealing temperature and extension time varied depending on the primer sets used in each reaction and the sizes of the expected PCR products.

PCR products were gel purified and ligated into the pMD18-T vector (TaKaRa, Dalian, China). At least three positive clones of each PCR product were sequenced at Shanghai Sangon Biological Engineering & Technology and Service Co. Ltd. (Shanghai, China). The obtained sequences by using RNA1-specific primers were assembled into contiguous sequences by overlapping common regions (>100 bp) of the amplicons.

### 2.4. Transmission Electron Microscopy (TEM) Observations

To study the distribution of virions in jujube cells, fresh young leaves of a symptomatic jujube plant were cut into approximate 4 mm × 2 mm slices and fixed in 2.5% (*w*/*v*) glutaraldehyde overnight. They were then rinsed twice with PB solution and fixed in 1% osmium tetroxide for 2 h, followed by ethanol gradient dehydration and embedding. Ultrathin sections were double-stained with 2% *w*/*v* lead citrate and 5% uranyl acetate, and examined with a TEM (H-7000 FA, Hitachi, Tokyo, Japan) [23].

### 2.5. RT-PCR Detection of the JYMaV

To develop a robust detection system, four pairs of primers were designed according to the sequences of the viral RNA3-RNA6 and used for the simultaneous amplifications of these RNAs (Table 1). In the growing seasons in 2018 and 2019, a total of 85 leaf samples were collected from jujube trees. These samples consisted of 61 samples from symptomatic jujube trees and 14 samples from asymptomatic jujube trees grown at Aksu region in Xinjiang Uygur Autonomous Region of China, and 10 samples from asymptomatic jujube trees in Shanxi Province. A leaf sample of a healthy jujube plant from Hubei Province was used as a negative control. Total RNA extractions and reverse transcription (RT) were done as described above. The presence of JYMaV in the samples was tested by RT-PCR assays using three pairs of primers targeting to the virus RNA3, RNA5 and RNA6. PCR products were separated by electrophoresis on 2% agarose gels, stained with ethidium bromide, and visualized under UV light.

### 2.6. Sequence Analyses

ORFs were predicted using NCBI ORFfinder (https://www.ncbi.nlm.nih.gov/orffinder/, accessed on 17 July 2020) with minimal ORF length of 75 nt. The potential cleavage sites, glycosylation sites, and transmembrane helices were predicted using SignalP 4.1 (http://www.cbs.dtu.dk/services/SignalP/ accessed on 17 July 2020), NetNGlyc Server 1.0 (http://www.cbs.dtu.dk/services/NetNGlyc/ accessed on 17 July 2020), and TMHMM Server 2.0 (http://www.cbs.dtu.dk/services/TMHMM/ accessed on 17 July 2020) with the default parameters, respectively. The potential nuclear localization signal was predicted using cNLS Mapper with a cut-off score 2.0 [24]. Sequence identities were computed using LALIGN program in EMBL-EBI (https://www.ebi.ac.uk/Tools/psa/lalign/ accessed on 17 July 2020) with the default settings. For phylogenetic analysis, multiple sequence alignments were performed using the Muscle program in MEGA 7.0 software [25], then phylogenetic trees were generated by using the Neighbor-Joining (NJ) method with a p-distance model and 1000 bootstrap.

### 2.7. Subcellular Localization and BiFC Analysis

For protein localization and bimolecular fluorescence complementation (BiFC) assays, the JYMaV ORF4 and ORF5 (without stop codon) were amplified using specific primers flanked with an *att*B recombination sequence (Table S1). The amplicons were gel-purified, and cloned into pDONR-ZEO using BP Clonase™ II Enzyme Mix (Invitrogen, Carlsbad, CA, USA). Sequence-validated entry clones were recombined into each of two split-YFP binary vectors (pEarlygate202-YCand pEarlygate201-YN) for BiFC assays and into pEarlygate101-YFP for subcellular localization experiments, respectively, using the LR Clonase™ II Enzyme Mix (Invitrogen). All the constructs were transformed individually into *Agrobacterium tumefaciens* strain GV3101 (Weidi Bio, Shanghai, China). The CMV3a-mCherry and H2B-mCherry vectors were used as plasmodesma (PD) and nuclear markers, respectively. Agroinfiltration methods to determine intracellular localization and interactions of the two proteins P4 and P5 encoded in JYMaV RNA4 and RNA5 were the same as reported previously [26]. Florescence signals of fusion proteins transiently expressed in leaves of *N. benthamiana* plants (5 weeks old) were visualized using confocal laser scanning microscopy (CLSM; TCS-SP8, Leica Microsystems, Heidelberg, Germany) with an HC PL APO CS2 63x/1.20 WATER objective.

## 3. Results

### 3.1. Jujube Viruses Identified by Using sRNA-Seq and RNA-Seq

Initially, 44,533,809 raw sRNA reads were obtained from the sample HZ. After removing adaptor sequences and filtering for quality, 39,607,094 clean reads were *de novo* assembled using Velvet. The resulting contigs were subjected to BlastX and BlastN searches against the nr and nt databases at the NCBI. Totally, 90 contigs assembled from the sRNA library were homologous to P1, P2, P3, P4 and P8 of raspberry leaf blotch virus (RLBV), a member of the genus *Emaravirus* (Table S2). When the genomic sequence of JYMaV was reported and available in NCBI GenBank [7], sequences alignment showed that these assembled emaravirus contigs well matched the genomic RNAs of JYMaV. Additionally, 26 contigs with length 131–464 nt matched the proteins of JuMaV with identities of 69.6–93.9%.

In total, 83,968,748 and 68,691,440 RNA reads were obtained from the samples AKS-6 and AKS-15, respectively. After filtering for read quality and sequence assembling, 154,611 and 133,053 contigs were generated from AKS-6 and AKS-15 libraries, respectively. BlastX and BlastN searches against the nr and nt databases at the NCBI revealed that six contigs respectively generated from the AKS-6 and AKS-15 libraries were homologous to genome RNAs 1–6 of JYMaV (Table S3), respectively. Additionally, some contigs from the two libraries matched the proteins of JuMaV [6] and potential new viruses. Here, only sequences of JYMaV were included in the following analyses.

The Raw fastq files of the sRNA-Seq library of HZ and RNA-Seq libraries of AKS-5 and AKS-6 were deposited in the GenBank with an SRA number PRJNA684042.

### 3.2. Genomic Sequence of the Three JYMaV Isolates

**Figure 1:**
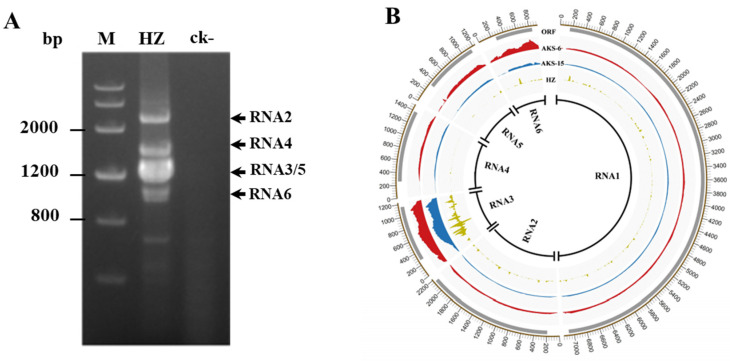
Electrophoresis analysis of RT-PCR products of the genomic RNA segments of jujube yellow mottle-associated virus (JYMaV) in the sample HZ by using the primer set 3C/5H (**A**) and profile distribution of sRNA reads of isolate HZ and RNA reads of two isolates AKS-6 and AKS-15 along genomic RNAs 1–6 of JYMaV-HZ (**B**). ck-, a leaf sample of a healthy jujube plant from Hubei Province. M, DNA marker Ⅲ. ORF in each RNA segment of JYMaV was shown by using a grey line above the read depth graphic in panel B.

RT-PCR using primer set 5H/3C revealed five amplicons from the sample HZ (Figure 1A). Cloning and sequencing of the RT-PCR products permitted to identify the five RNA segments named RNA2, RNA3, RNA4, RNA5 and RNA6 (two clones with different sizes, name RNA6-1 and RNA6-2). RNA6-1 and RNA6-2 were the two bands close to each other with a size about 940 bp, which had highly similar sequences. Meanwhile, RNA1 was sequenced by overlapping RT-PCR assays using primers designed based on the contig sequences from sRNA-Seq data. Then, six genomic RNA components of JYMaV were identified from the sample HZ.

The RNA sequences obtained by Sanger sequencing of RT-PCR products from HZ and the near complete sequences of JYMaV genomic RNAs 1–6 from the datasets of AKS-6 and of AKS-15 had the sequence features of emaravirus RNAs, with each RNA containing a single ORF on its complementary-sense RNA (cRNA). The 5′ and 3′ terminal sequences of RNA3 determined by RACE analysis harbored 13-nt complementary stretches of 5′-AGUAGUGUUCUCC-3′ at its 5′ terminus and 5′-AGUAGUGAACUCC-3′ at its 3′ terminus, a typical feature of members in the genus *Emaravirus* [27,28]. The sizes of untranslated regions (UTRs) of the JYMaV-HZ RNAs ranged from 150 nt (RNA1) to 314 nt (RNA5) for their 5′ terminus and 57 nt (RNA2) to 101 nt (RNA4) for their 3′ terminus. The terminal nucleotides of each RNA segment of JYMaV genome could form a complementary structure containing a conserved (1–13 nt) and a variable region (Figure S1). The nucleotide sequences of RNAs 1–6 of JYMaV isolates HZ, AKS-6 and AKS-15 were submitted to GenBank with accession numbers MT953011–MT953017, MT953018–MT953023, and MT953024–MT953029, respectively.

The ORF1 of three isolates HZ, AKS-6 and AKS-15 from Aksu jujube samples consisted of 6930 nt, encoding a protein (RdRp, P1) with a predicted molecular weight (MW) of 271.1 kDa. Pairwise comparisons showed that the ORF1 of these isolates shared about 93.3–98.2% nt and 97.3–99.4% aa sequence identities with each other and 93.3–98.6% nt and 97.2–99.4% aa sequence identities with that of isolate SY (Table 2). P1 harbored all motifs conserved in members of *Bunyavirales* and an endonuclease domain H_118_-D_141_-PD_157–158_-EVK_172–174_ [29].

The RNA2 sequences were mostly variable among the genomic RNA segments of JYMaV. The ORF2 size of the JYMaV isolates HZ, AKS-6 and AKS-15 was 1974 nt, which was 12-nt shorter than 1986-nt ORF2 of isolate SY. The difference was caused by a 9-nt deletion at position 2049–2057 nt and a 3-nt deletions at site 2066–2068 nt in the RNA2 of Aksu JYMaV isolates. The ORF2 of HZ, AKS-6 and AKS-15 shared 92.8–95.7% nt and 94.7–97.3% aa sequence identities with each other, and 88.0–89.6% nt and 90.7–91.8% aa sequence identities with isolate SY (Table 2). The glycoprotein precursor (GP, P2) encoded in the ORF2 of HZ, AKS-6 and AKS-15 consisted of 657 aa with a predicted MW of 75.8 kDa. The protein contained essential sequence elements of emaravirus GPs, including one N-terminal signal peptide, four transmembrane helices, four putative glycosylation sites and two cleavage sites. Two cleavage sites at VLA_216_↓D_217_D and TYS_23_↓S_24_V were predicated to cleavage the GP into Gn (22.2 kDa), Gc (50.9 kDa), and Gs (2.7 kDa).

The ORF3 of these isolates had the same size of 885 nt as that of isolate SY and encoded a 294-aa nucleocapsid protein (NP, P3) with a predicted MW of 33.1 kDa. It contained three blocks (NVVSYNR_130–136_, NKLA_177–180_ and GFEF_198–201_), which were conserved in the NP of reported emaraviruses [16,19,29]. The ORF3 of HZ, AKS-6 and AKS-15 shared 98.6–98.9% nt and 98.3–99.0% sequence aa identities with each other, and 95.5–95.9% nt and 94.9–95.2% aa sequence identities with isolate SY (Table 2).

The ORF4 of the three JYMaV isolates was 1131 nt, which was 6-nt longer than that (1125 nt) of isolate SY. Sequence alignments revealed that the ORF4 of these isolates had two 3-nt deletions at positions 998–1000 nt and 1049–1051 nt, and a 12-nt insert at position 1390nt as compared to isolate SY. The ORF4 of the three JYMaV isolates encoded a movement protein (MP, P4) consisted of 376 aa with a predicted MW of 42.5 kDa. Pfam analyses revealed that the P4 had typical features of the 30K superfamily of plant virus MPs [30]. The ORF4 of the three JYMaV isolates shares 95.2–96.8% nt and 99.5–100% aa sequence identities with each other, and 91.5–91.8% nt and 95.4% aa sequence identities with isolate SY (Table 2).

The ORF5 of the three Aksu isolates and isolate SY were 837 nt and encoded a protein (P5) consisted of 278 aa with a predicted MW of 32.0 kDa. The ORF5 of the three Aksu JYMaV isolates shares 92.6–97.8% nt and 92.4–97.1% aa sequence identities with each other, and 92.7–94.7% nt and 92.4–95.7% aa sequence identities with isolate SY (Table 2). Further sequence analyses revealed that the P5 contained two bipartite NLSs (aa positions: 2–50 and 225–256), and also the secondary structural elements of the 30K superfamily of plant virus MPs.

RNA6 determined from HZ by RT-PCR using primer set 5H/3C had two lengths of 941 nt and 983 nt. The major difference between the two sequences occurred at their 5′UTRs, which had divergent lengths of 267 nt and 309 nt and were 82.2% identical to each other. The ORF6 sequences of the two RNA6 variants (RNA6-1 and RNA6-2) from HZ were 585 nt and 96.8% identical to each other at nt level. The protein (P6) encoded by the two RNA6 variants consisted of 278 aa with a predicted MW of 23.0 kDa, and were 96.4% identical to each other. The ORF6 size of isolates AKS-6 and AKS-15 was the same as that of isolates HZ and SY. All ORF6 sequences from the three Aksu samples HZ, AKS-6 and AKS-15 shared 91.6–97.9% nt and 91.8–97.4% aa sequence identities with each other, and 94.2–94.5% and 93.3–94.3% aa sequence identities with isolate SY (Table 2). The viral P6 displayed low aa sequence similarities of 36.4%, 26.3% and 42.8% with RLBV proteins P6, P7 and P8, but not with proteins encoded by other reported emaraviruses and proteins reported in GenBank.

### 3.3. Distribution of JYMaV-Derived sRNA and RNA Reads along the Viral Genome

In total, 2,028,384 (accounting for 0.051% of total sRNAs) sRNAs of JYMaV derived from sample HZ, and 989,922 (accounting for 0.012% of total reads) JYMaV-RNA reads from sample AKS-6 and 1,329,988 JYMaV-RNA reads (accounting for 0.02% of total reads) from AKS-15 were mapped to gRNA sequences of JYMaV-HZ. When RNA reads deriving from JYMaV isolates AKS-6 and AKS-15 were individually mapped onto six RNAs of JYMaV-HZ genome, it was found that the two isolates showed similar read distribution profiles on the viral RNAs (Figure 1B). RNA reads covered the full lengths of the viral RNAs 1–6. Prevalent read peaks were observed in the RNA3 and RNA6 of the two isolates, followed by RNA5 and RNA4. In consistent with it, high sRNA read depth from JYMaV-HZ presented in RNA3 and a clear peak was also observed in RNA6. The sRNA read depths in the viral RNA4 and RNA5 were very low (Figure 1B).

### 3.4. Simultaneous Amplification JYMaV RNA Segments

**Figure 2:**
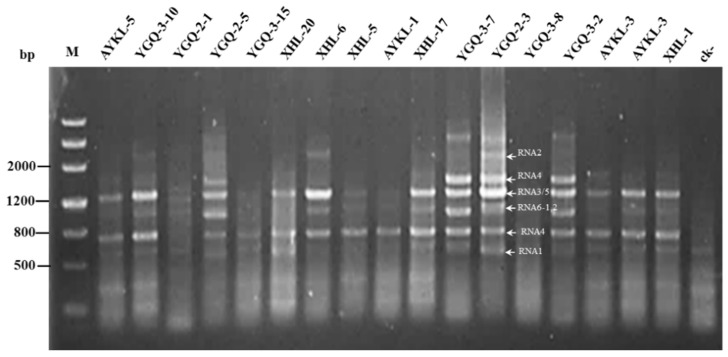
Electrophoresis of RT-PCR products obtained from 17 jujube samples positive to jujube yellow mottle-associated virus (JYMaV) by using the primer set 5H/3C. ck-, a leaf sample of a healthy jujube plant from Hubei Province. M, DNA marker Ⅲ. Bands of RT-PCR products from sample YGQ-2–3 were marked with the viral RNAs basing on their sequences.

To make sure whether JYMaV had additional RNA segments and to view sequence diversity of JYMaV RNA segments, we performed RT-PCR assays for 17 diseased jujube samples using primer set 5H/3C. Electrophoretic analysis of the PCR products from these samples revealed that four bands corresponding to the genomic RNAs 2, 3/5, 4 and 6 of JYMaV were recovered from samples YGQ-2-3, YGQ-2-5, YGQ-3-2, YGQ-3-7 and YGQ-3-10 and two or three bands corresponding to the viral RNAs 4 and 6 or 3/5, 4 and 6 were amplified from additional samples (Figure 2). For the sample YGQ-2-3, near the position of RNA6, there were obviously two very close bands. Cloning and sequencing revealed that they were two products of RNA6 with divergent lengths (941 nt and 991 nt) as illustrated for that from the sample HZ. Visualization for the RNA6 product bands amplified from different samples showed that the viral RNA6 might be slightly variable in sizes. The band above RNA2 band was a product from host genome as confirmed by sequencing. Additionally, two products between the 800 bp and 500 bp marker bands were amplified from most samples. Sequencing for the products revealed that they were a fragment (706 bp) of the viral RNA4 and a fragment (525 bp) of the viral RNA1, respectively. These bands were not amplified from a negative sample. Then, RT-PCR using primer set 5H/3C did not identified additional RNA segments of JYMaV, confirming that the JYMaV genome consisted of six RNA segments.

**Figure 3:**
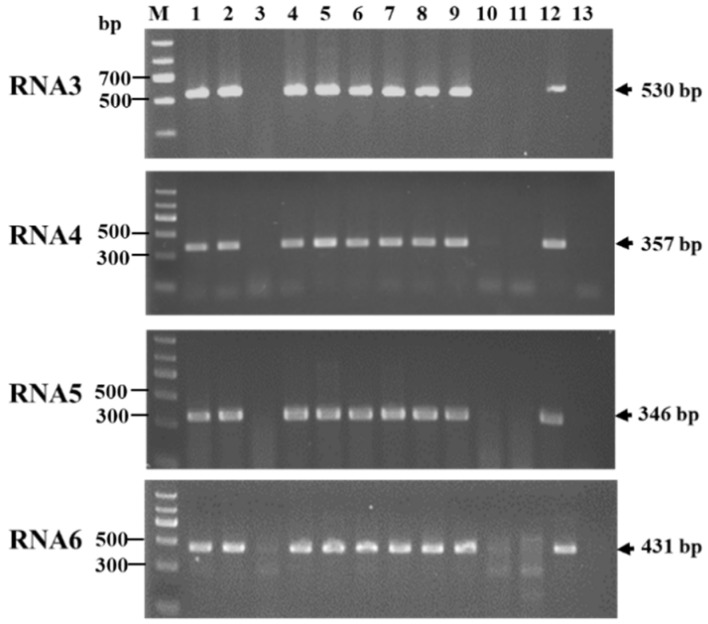
RT-PCR detection of four RNA segments (RNAs 3–6) of jujube yellow mottle-associated virus (JYMaV) in 11 jujube samples from Aksu using four pairs of primers. The sizes of amplicons were indicated on the right of each panel. The sample HZ tested by high-throughput sequencing was used as a positive control (line 12) and a leaf sample from a healthy jujube plant was used as a negative control (line 13). M: DNA marker II.

Furthermore, four pairs of primers were designed based on the sequences of the viral RNAs 3–6 and used for the RT-PCR detection of JYMaV in 13 jujube samples. Amplicons with expected sizes were detected in nine diseased jujube samples (including the sample HZ tested by sRNA-Seq), but not in the three asymptomatic samples from Xinjiang and one sample (negative control) from Hubei Province (Figure 3). The results strongly supported the association of these RNAs with the virus infection and JYM disease.

### 3.5. Sequence Diversity of RNA Segments among JYMaV Isolates

Sequencing RT-PCR products obtained using primer set 5H/3C revealed sequence diversity among different JYMaV isolates, particularly in the extended non-coding regions of the viral RNAs 1, 4, 5, 6, as illustrated as followings. The sequences amplified using 3C/5H were submitted to GenBank with an accession number MT975909 for RNA2 of XHL-17, and accession numbers MT975910–MT975921 for RNA3 of AKS-5, AKS-9, AKS-18, AKS-20, AYKL-5, GS, XHL-5, XHL-6, XHL-17, XHL-20, YGQ2-3 and YGQ3-7, and MT975922 and MT975923 for RNA4 of YGQ2-3 and YGQ3-7, and MT975924–MT975928 for RNA5 of AKS-5, AYKL-3, YGQ2-3 and YGQ3-7, and MT975929–MT975932 RNA6 of isolates YGQ2-3-4, YGQ2-3-2, YGQ2-5, YGQ3-7 and AKS-5.

A fragment about 522 bp and a fragment about 1600 bp at the 3′ end of the viral genomic RNA1 were amplified from samples AKS-20, YGQ2-3, YGQ2-5 and AKS-18, and samples YGQ3-10, YGQ2-1, XHL-17 and AYKL-5, respectively. Multiple alignments of these fragment sequences together with the corresponding sequences of isolates HZ, AKS-6, AKS-15 and SY showed that a 21-nt sequence (5′-UGC/UUGU/CAACUGUGUUCCGUUU-3′) located at about 62 nt from the 3′-terminus of the viral RNA1 repeated in some isolates (Figure S2). Isolates HZ, AKS-20, YGQ2-3 and YGQ2-5 harbored one copy of the sequence. The sequence appeared twice in isolates AKS-18 and SY and even three times in isolates YGQ3-10, YGQ2-1, AYKL-5, AKS-6 XHL-17. However, other regions of the viral RNA1 showed very low sequence variation among these isolates.

The viral RNA2 sequence was obtained by using primer set 5H/3C from the sample XHL-17. The sequence showed high identity of 93.2–99.6% to the corresponding sequences of isolates HZ, AKS-6, AKS-15, and was 88.0% identical to that of isolate SY. The ORF2 size of XHL-17 was the same as that of isolates HZ, AKS-5, AKS-16.

The complete sequences of the viral RNA3 were obtained from 12 samples by using the primer set 5H/3C. These sequences were 1225–1334 nt in length and 98.2–99.8% identical to that of isolate HZ and 95.5–96.3% identical to that of isolate SY. The ORF3 encoded in all these sequences was 885 nt. The 5′ UTR of all JYMaV isolates from Aksu jujube samples had a 29-nt deletion (5′- AGUAUUGUAAUACAAUGAAGCAUAAAAUA-3′) at position 63–91 nt near 5′ terminus of the viral genomic RNA3 when it was compared with that of isolate SY (Figure S3). Additionally, it was found that nucleotide A was enriched in the 5′ UTR of the viral genomic RNA3.

The complete sequences of the viral RNA4 were obtained from samples YGQ2-3 and YGQ3-7 by using the primer set 5H/3C. RNA4 clones from the two samples had the same size of 1493 nt as that of isolate HZ. The viral RNA4 sequences obtained from Aksu jujube samples shared over 93.3% nt identity with each other and 89.9–90.9% nt identity with SY. The ORF4 of YGQ2-3 and YGQ3-7 was 1131 nt, which was the same as that of isolates HZ, AKS-5, AKS-16 and was 6-nt longer than that (1125 nt) of isolate SY. The 5′ UTR sizes of the viral RNA4 were highly variable among JYMaV isolates. Except for AKS-15 having a 8-nt insert at sites 94–101 nt as that in SY, the 5′ UTR of all RNA4 clones of Aksu isolates had four deletions at sites 94–101 nt, 176–199, 262–274, and 288–300 nt as it was compared with that of isolate SY (Figure S4).

The complete nucleotide sequences of the viral RNA5 were obtained from four samples AKS-5, AYKL-3, YGQ2-3 and YGQ3-7 by using the primer set 5H/3C. The viral RNA5 sequences obtained from Aksu jujube samples shared over 92.2% nt identity with each other and 91.2–93.7% nt identity with SY. The ORF5 of all available isolates had the same size of 837 nt. However, the 5′ UTR lengths of the viral RNA5 were highly variable among isolates, ranging from 314 nt to 354 nt (Figure S5).

The complete nucleotide sequences of the viral RNA6 were obtained from four samples AKS-5, YGQ2-3, YGQ2-5 and YGQ3-7 by using the primer set 5H/3C. The analysis of RNA6 sequences gave a more complicated result. Like isolate HZ, two divergent RNA6 variants were sequenced from isolate YGQ2-3. Of five RNA6 clones from isolate YGQ2-3, four clones had similar sizes of 942 or 944 nt and one clone was 991 nt, which was in consistent with the two RNA6 bands of PCR products amplified using 3C/5H from the sample. These RNA6 clones of isolate YGQ2-3 shared 92.1–100% nt sequence identity and 90.2–100% aa sequence identity for ORF6 (P6). Four RNA6 clones from isolate AKS-5 were 956 nt. Clones of RNA6 from isolate YGQ2-5 and YGQ3-7 were 940–944 nt. RNA6 clones from these samples together with isolates HZ, AKS-6, AKS-15 shared 88.8–99.8% nt sequence identity with each other and 91.1–93.5% nt sequence identity with SY. Multiple alignments of all RNA6 sequences from Aksu isolates and isolate SY revealed that in addition to nt and aa variations located across the ORF6, notable sequence diversity happened at the 5′ UTR with lengths ranging from 267 to 319 nt (Figure S6) and identities ranging from 81.6% to 89.2%. However, the ORF6 size (585 nt) encoded in these sequences was the same.

**Figure 4:**
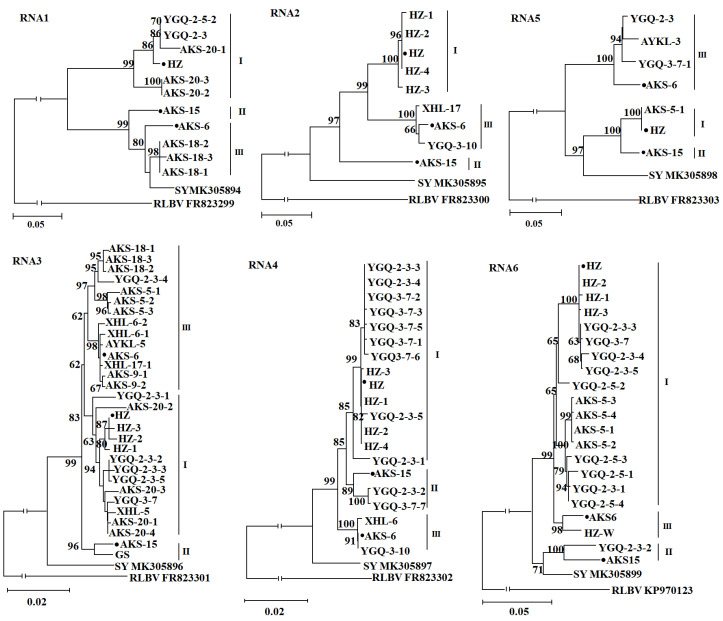
Unrooted neighbor joining (NJ) phylogenetic trees generated from the nucleotide sequences of RNAs 1–6 of jujube yellow mottle-associated virus (JYMaV). In the RNA1-based tree, fragments (525–1619 nt at 3′ end of RNA1) obtained from samples AKS18, AKS20, YGQ-2-3 and YGQ-2-5 by RT-PCR using primer set 5H/3C were included in the assay. The sequence of reported isolate SY in each tree was marked by its isolate name followed by a GenBank accession number. Three JYMaV isolates determined by high throughput sequencing were highlighted by black dots. The corresponding RNA sequence of raspberry leaf blotch virus (RLBV) was used as an outgroup in each phylogenetic tree.

In phylogenetic trees constructed using the complete nucleotide sequences of JYMaV RNAs 2–6 and partial sequences at the 3′ end of the viral genomic RNA1 showed that JYMaV variants from Aksu jujube samples clustered as three clades represented by isolates HZ, AKS-15 and AKS-6, and the isolate SY from Liaoning Province in China always stayed as a separate clade (Figure 4). Notably, clones of isolate YGQ-2-3 were sequence divergent and distributed in different clades in each of the RNA3-, RNA4- and RNA6-based trees. In the RNA6-based tree, out of five clones of isolate HZ, one clone (HZ-W) was separated from other four clones in clade I. Similarly, in the RNA4-based tree, the clone YGQ-3-7-7 was phylogenetically separated from other five clones from the same sample YGQ-3-7. The results suggested that these jujube plants might be co-infected by molecularly divergent JYMaV variants. These results further confirmed the molecular diversity within the Aksu JYMaV populations.

### 3.6. Subcellular Localization and Interaction of JYMaV P4 and P5

**Figure 5:**
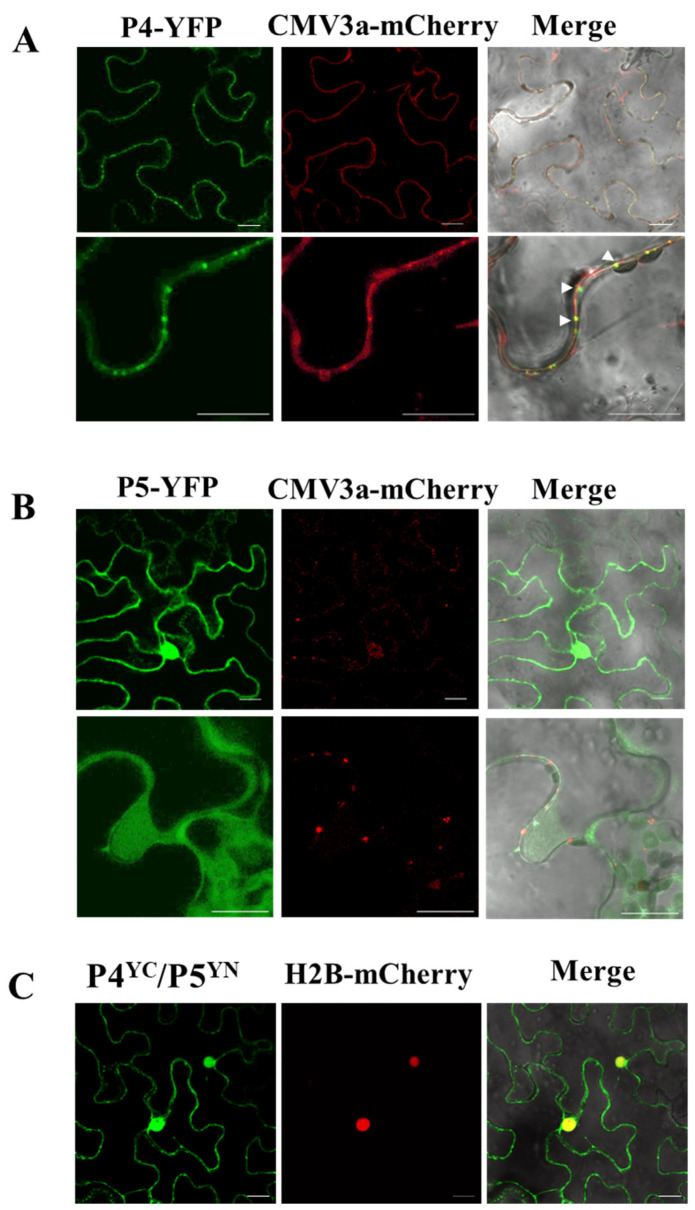
Subcellular localization (**A**,**B**) and bimolecular fluorescence (BiFC) (**C**) assays of P4 and P5 of jujube yellow mottle-associated virus (JYMaV) in epidermal cells of wild-type *Nicotiana benthamiana* leaves. The fusion proteins CMV3a-mCherry and H2B-mCherry were used as plasmodesmata and nucleus markers, respectively. Colocalization dots of JYMaV-P4 with CMV3a-mCherry at PD were indicated by arrow heads. Images were acquired 3 days after agroinfiltration under a confocal microscope at 63x/1.20 WATER objective. Scale bar = 20 μm.

The P5 sequence of JYMaV partially matched the viral P4, and there was notable aa sequence similarity (a whole aa sequence identity of 35.3%) between the two proteins. The predicated secondary structures of the two proteins were also similar, then it was postulated that as P4, the P5 might play roles in the virus movement [7]. To have a primary look at the function of the P5, the subcellular localizations of the viral P4 and P5 in epidermal cells of *N. benthamiana* leaves were investigated by using an *Agrobacterium* infiltration method. It was found that the two proteins distributed at different locations in epidermal cells of *N. benthamiana* leaves. At three days post infiltration (dpi), the fluorescence signals of diffused protein P4-YFP were observed as punctate spots along the cell membrane and co-localized with the plasmodesma (PD) marker protein CMV-3a-mCherry at PD in the epidermal cells (Figure 5A), indicating that the viral P4 showed a subcellular localization feature typical for the movement proteins of plant viruses. Whilst, the fluorescent signals of diffused protein P5-YFP located in cytoplasm and the nuclear periplasm, and did not co-localize with the PD marker (Figure 5B). The distribution profiles did not change at 5–6 dpi. To understand whether the two proteins could interact with each other, BiFC assays were carried out (Figure 5C). Fluorescence signals were observed in the leaf cells co-infiltrated with the paired proteins P4^YC^/P5^YN^, indicating the occurrence of interaction between the two proteins. Their interaction fluorescent signals located along cell membranes and in nuclear periplasm, which were in consistent with the subcellular localization signals of the viral P5. The result suggested that the P5 could affect the subcellular localization of P4 by recruiting P4 into nucleus.

### 3.7. Virus Particle Identification by TEM

**Figure 6:**
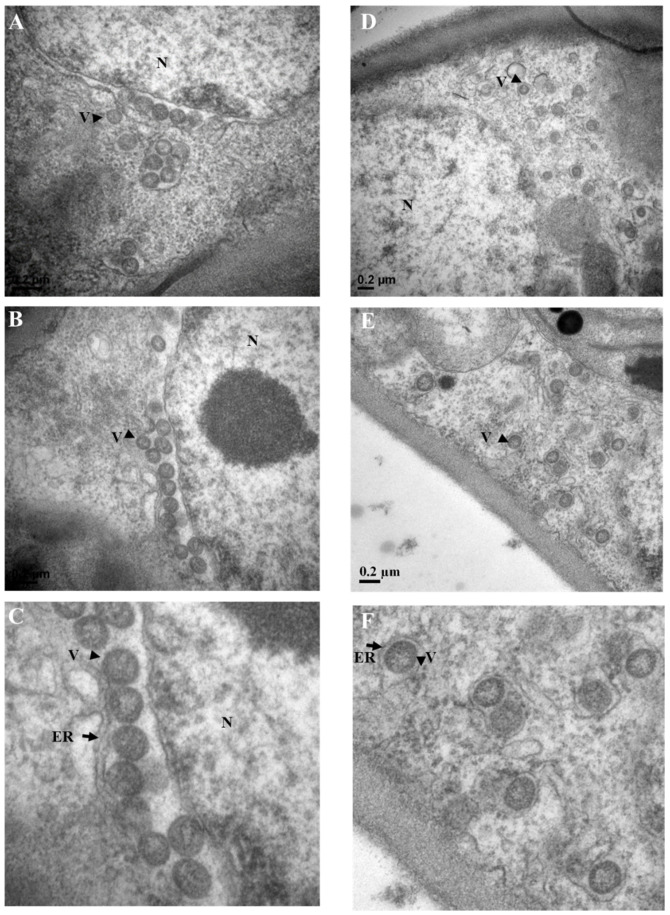
Transmission electron microscopy observation of jujube cells infected by jujube yellow mottle-associated virus (JYMaV). Spherical virus-like particles located between nucleus membrane and endoplasmic reticulum (**A**,**B**), aggregated in the cavity structure of endoplasmic reticulum (**A**) and individually surrounded by endoplasmic reticulum-like membrane structures in cytoplasm (**C**,**D**). The enlarged sections of (**B**,**E**) were shown in (**C**,**F**). Virus particles were indicated by arrow heads and endoplasmic reticulum-like structures were indicated by arrows. ER: endoplasmic reticulum; N: nucleus; V: virion. Scale bar = 0.2 μm.

Transmission electron microscopy observation revealed the presence of spherical virus-like particles (VLPs) of approximately 80–100 nm in diameter in negatively stained ultrathin sections of leaves from a symptomatic jujube tree (Figure 6). VLPs aggregated in cavity structures formed by nucleus membrane and/or endoplasmic reticulum in the cytoplasm (Figure 6A–C) or individually surrounded by membrane structures and dispersedly located in cytoplasm (Figure 6D–F). The VLPs were not observed in the petiole tissues of the symptomatic jujube tree and in the tissues of an asymptomatic jujube tree. The results supported the infection of JYMaV in mesophyll cells of the diseased jujube trees.

### 3.8. Occurrence of Jujube Yellow Mottle Disease and RT-PCR Assays for JYMaV

**Figure 7:**
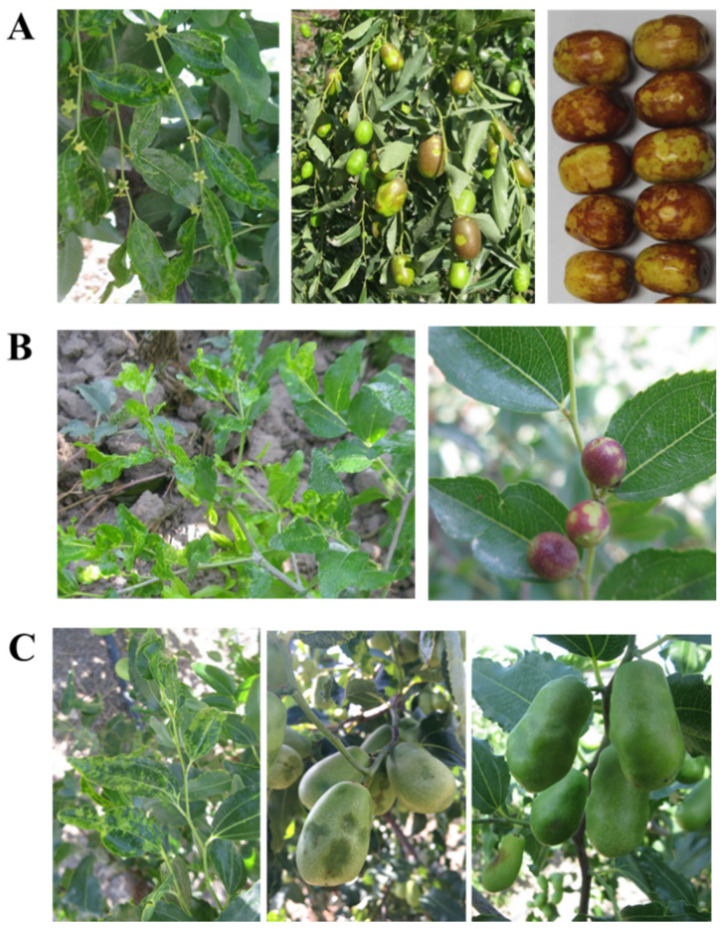
Disease symptoms on leaves and fruits of *Ziziphus jujube* cv. ‘Huizao’ (**A**), *Z. jujuba* var. ‘spinosa’ (**B**) and *Z. jujuba* cv. ‘Junzao’ (**C**).

During our field investigations carried out in 2017 to 2019, viral disease-like symptoms were observed on leaves and fruits of jujube trees grown at Aksu, in the Xinjiang Uygur Autonomous Region of China. The diseased trees of *Z. jujuba* cv. ‘Huizao’ showed severe yellow mottle and chlorotic spots on young leaves and produced dapple fruits (Figure 7A). The trees of *Z. jujuba* var. ‘spinosa’ growing at fields with severely diseased trees of *Z. jujuba* cv. ‘Huizao’ also showed yellow mottle and chlorotic spots on young leaves and produced dapple fruits (Figure 7B), which were highly like that on ‘Huizao’ trees. The leaves of diseased trees of *Z. jujuba* cv. ‘Junzao’ exhibited chlorotic spots or mottle on leaves, and dark-green spots on fruits and serious fruit malformation (Figure 7C). The severely diseased leaves of these trees became distorted along leaf edges.

To understand the incidence of JYMaV and its association with the observed disease, 85 jujube leaf samples, including 61 symptomatic leaf samples, 14 asymptomatic leaf samples from Aksu area in Xinjiang Uygur Autonomous region of China and 10 asymptomatic leaf samples from Shanxi Province, were subjected to RT-PCR assays for JYMaV using three primer sets 3-F/3-R, 5-F/5-R and 6-F/6-R. Results showed that JYMaV was exclusively detected in the jujube leaf samples showing chlorotic leaf spots (Table S4). Out of 61 symptomatic leaf samples, 55, 56 and 53 samples were positive for JYMaV as tested using primer sets 3-F/3-R, 5-F/5-R and 6-F/6-R, respectively (Table 3). For 61 symptomatic leaf samples, one sample AKS-12 was JYMaV-positive as tested using the primer set 3-F/3-R, but was JYMaV-negative as tested using the primer sets 5-F/5-R and 6-F/6-R, two samples XHL-3 and XHL-9 were JYMaV-negative as tested using the primer set 3-F/3-R, but were JYMaV-positive as tested using the primer sets 5-F/5-R and 6-F/6-R, and three samples were JYMaV-negative in tests using the three primer sets. All asymptomatic samples were negative for the virus.

## 4. Discussion

The reported JYMaV genome consisted of six negative-sense RNAs [7]. In this study, the full-length sequences of the six genomic RNAs of JYMaV infecting a jujube plant (cv. ‘Huizao’; ID. HZ) grown at Aksu in Xinjiang were determined by NGS analyze for sRNAs combined with Sanger sequencing. Meanwhile, the near full-length genomic RNA sequences of two JYMaV isolates were determined by individual NGS analysis for rRNA-deleted RNAs derived from two plants of jujube cv. ‘Junzao’ and var. ‘spinosa’. All these analyses identified six RNA segments with each containing one ORF in its antisense. The result indicated that JYMaV identified from Aksu jujube plants had the same genomic structure as that of JYMaV isolate SY [7]. The reported emaraviruses exhibit flexibility for the constitute of their genomic RNA segments [8]. Several emaraviruses contain up to eight and even ten different genome segments [12,17,18]. We did not identify any other RNA of the virus from diseased jujube samples by using different approaches, indicating that RNAs 1–6 might represent the complete genomic components of JYMaV. Prevalent peaks of RNA and sRNA reads on the RNA3 and RNA6 of JYMaV are highly similar to that of pear chlorotic leaf spot-associated virus (PCLSaV), a recently identified emaravirus from pear [31]. Similarly, high levels of vsiRNAs were generated from pigeonpea sterility mosaic emaravirus 1 (PPSMV-1) RNA3 and RNA4 segments [32]. The distribution profiles might be related to the genome expression strategy of the viruses since the transcription of viral mRNAs is the first step to encode proteins with specific functions. Accordingly, the high level of mRNAs could provide templates for the targeting by the Dicer-like proteins to generate sRNAs [33].

RT-PCR assays using primer set 5H/3C allowed the full-length amplification of JYMaV RNAs (except for RNA1) from different jujube samples as that used for some other emaraviruses [12,19,28,34]. Meanwhile, primer set 5H/3C also amplified fragments of the viral genome RNA1 and RNA4, but not additional genomic RNA segments of the virus. The fragments showed very high identities with the corresponding regions of known JYMaV RNA segments and absence of full ORFs and the typical terminal stretches of emaravirus genomic RNAs. Comparison of genomic RNA sequences of JYMaV obtained by RNA-Seq and 5H/3C-based RT-PCR assays revealed significant sequence variability among the JYMaV isolates from Aksu jujube plants and the isolate SY. The RNA6 was the most divergent among all JYMaV RNA segments. Our study revealed the presence of a population of RNA6 molecules, which had high sequence diversity in their 3′ UTR. This RNA6 sequence heterogeneity was supported by two different sizes of 5H/3C-primered RT-PCR products of RNA6 in a single plant. Genetic variations have been reported among isolates of some emaraviruses, including fig mosaic emaravirus (FMV), High Plains wheat mosaic virus (HPWMoV), PPSMV-1, pigeonpea sterility mosaic emaravirus 2 (PPSMV-2) and redbud yellow ringspot associated virus (RYSaRV) [15,35,36,37]. HPWMoV isolates from Nebraska and Kansas harbor two variants of RNA 3 (3A and 3B) with 11% amino acid differences [11]. However, the sequence heterogeneity of JYMaV RNA6 did not affect encoded protein (P6) sequence, which was different from the heterogeneity RNAs encoding heterogeneity proteins of some other emaraviruses [36]. The RNA3 sequences of JYMaV were more conserved than other RNA sequences and shared about 98–99% nt identity among Aksu isolates, which was different from PPSMV-1 and PPSMV-2 isolates with more divergent RNA3 than RNA4 [37]. The variation of UTRs also presented in other RNA segments of the virus. Changes in the UTRs caused difference in the lengths of the viral genomic RNA segments among different isolates or clones. Particularly, sequences repetition occurred at the 3′ UTR of the viral RNA1 segment. The variation at the UTRs might be relevant for the ability of JYMaV to adapt specific selection pressures, then for its replication and pathogenicity [34,38]. Viral RNA synthesis requires specific interaction between the viral genomic RNA and the replicase. The 3′- and 5′-terminal regions of each segment of negative-sense RNA viruses are complementary and capable of forming a panhandle structure, which is one of the hallmarks of negative-sense multipartite ssRNA viruses and essential for viral transcription and replication [39,40,41,42]. The terminal nucleotides of JYMaV RNAs can form a complementary structure containing a conserved (1–13 nt) and a variable region, which is highly similar to that of bunyaviruses [43]. Whether the terminal interaction of JYMaV RNAs has functional feature of other segmented negative-sense RNA viruses is unknown. Phylogenetic analyses of the viral RNA segments revealed that these Aksu isolates were molecularly divergent from the isolate SY identified from a jujube plant grown at Shenyang, China [7], indicating that these JYMaV isolates from Aksu and isolate SY might differ in their origins. However, sequence analyzes did not reveal any recombination or RNA segment reassortment event in the obtained sequences, which has been reported for PPSMV-1, PPSMV-2 and FMV [35,37] The considerable genetic variability of JYMaV Aksu isolates suggested that the virus was not recently introduced to Aksu jujube plants. The factors causing the sequence variation of these viruses need to be investigated further.

RNA segments encoding for redundancy proteins have been reported in HPWMoV (P5 and P6) [11], RLBV (P6, P7, and P8) [12], rose rosette virus (RRV) (P5 and P7) [34,44], Pistacia virus B (PiVB) (P5) [17], European mountain ash ringspot-associated virus (EMARaV) (P4 and P6) [45], and Perilla mosaic virus (PerMV) (P6a, P6b, and P6c) [18]. Whether the homology proteins within an emaravirus species or isolate have synergistic effect is unknown. Similarly, a previous study suggested that both proteins P4 and P5 of JYMaV could be viral MPs due to their limited sequence similarity and harboring the typical feature of 30K MP superfamily [7]. Our study results showed that the viral P4 displayed PD subcellular localization, a typical feature of the movement proteins of plant viruses, but the viral P5 located in cytoplasm and the nuclear periplasm. BiFC assays showed the interaction between the two proteins changed the subcellular localization of P4 into nucleus. Further study on biological mechanism of the interaction may provide further insights into the P5 function. In addition, the JYMaV P6 showed low amino acid sequence similarities to RLBV proteins P6, P7 and P8, which play roles in RLBV pathogenicity [7,12]. Then, JYMaV P6 also might be a pathogenic factor.

RT-PCR analyses for leaf samples of diseased jujube plants provided a robust association of JYMaV with the jujube disease occurring at Aksu. For the first time, our results showed the JYMaV infection in different cultivars and one important jujube rootstock species widely grown at Aksu in China. Although the three sets of primers developed in this study could be used for the efficient RT-PCR detection of JYMaV, there were three symptomatic leaf samples to be JYMaV-negative in the RT-PCR assays. The reason might be that the infections of other viruses in jujube plants could induce similar symptoms as those caused by JYMaV [6]. The low titer or uneven distribution of the virus might also affect the detection [12]. In addition, during our field surveys at the growing season of jujube plants, we found wide infestation of eriophyid mites on jujube plants. RT-PCR tests also showed that eriophyid mite samples from diseased jujube plants were JYMaV positive, suggesting that eriophyid mites might vector JYMaV and cause the wide distribution of the viral disease at Aksu jujube fields. The high RNA-Seq read numbers within the ORFs of the viral RNA3 and RNA6 might be favorable for developing RNA3- and RNA6-based RT-PCR detection of JYMaV as that for other emaraviruses [31,46]. 

The extensive investigations at more regions are necessary for the further realization of the epidemiological tendency of the viral disease and molecular characteristics of JYMaV. Since the virus is prevalent at the Aksu area, it is necessary to minimize the impact of the viral disease by using certified materials for the propagation of plantlets.

## Figures and Tables

**Table 1:** Primers used for the amplification of full-length cDNAs of JYMaV RNAs from sample HZ and RT-PCR detection for JYMaV in jujube samples.

RNA	Primer	Sequence ^a^ (5′–3′)	Position	Product Size
			(nt)	(bp)
Amplification of full-length cDNAs of JYMaV				
RNA1	5H	CTCAGCAGTAGTGTTCTCC	1–13	1066
	1087R	AGTGCTATCATTCCACAGACC	1067–1087	
	690F	GACCCATTGCCTCTATGTTC	690–708	1539
	2228R	AAGATGTATACAATAAGATC	2209–2228	
	2072F	CTACTGATCCTTTGCCATTCC	2072–2092	1303
	3374R	GCTCTGCCAAATTAATGTCTG	3354–3374	
	AF	GATGCATCDAAATGGTCWGC	3283–3370	390
	CR	ATCATCWGARTGHACCAT	3653–3672	
	3438F	GACTATTTGTGGTATAATTC	3438–3457	1897
	5534R	CAGACAACTGATCCTAACAC	5315–5334	
	5199F	GGAGCTGGTCGAACTCTATG	5199–5218	1533
	6712R	GGCATGACATGATGATAGAT	6712–6731	
	6534F	GTTCTAGATCTGTCTTCATGGT	6534–6555	627
	3C	CTCAGCAGTAGTGAACTCC	7148–7160	
RNA2–6	5H	CTCAGCAGTAGTGTTCTCC	5′ end	-
	3C	CTCAGCAGTAGTGAACTCC	3′ end	-
5′RACE	Outer	CATGGCTACATGCTGACAGCCTA	-	652
	R3–5R1	CTTGCTTGTGGATTTGGTGAAG	597–618	
	Inner	CGCGGATCCACAGCCTACTGATGATCAGTCGATG	-	435
	R3–5R2	AGGTTATAGCAGATCTTGGGAC	389–410	
3′RACE	Outer	TACCGTCGTTCCACTAGTGATTT	-	483
	R3–3F1	CGCACCTGGCTTAACCTTAG	778–797	
	Inner	CGCGGATCCTCCACTAGTGATTTCACTATAGG	-	290
	R3–3F2	GGGATACTGTTGCCAAGTTGAG	952–971	
RT-PCR detection for JYMaV				
RNA3	3-F	CTTCACCAAATCCACAAGCAAG	597–618	530
	3-R	CACATCACAGAAAAATGAGACC	1105–1126	
RNA4	4-F	GAGCAGTCCACATTAACTAAG	887–907	357
	4-R	GGAATCTGGATCAAAGAACAC	1223–1243	
RNA5	5-F	GCCCTTATTCCTATTCTCATCA	744–765	346
	5-R	ATCAAGTTCGAGAAGTGTGG	1070–1089	
RNA6	6-F	CAAGCATAGCAATTGATGAC	387–406	431
	6-R	ACTTGATCTGTGGACTGAAC	798–817	

^a^ D: G/A/T, W: A/T, R: A/G, H: A/T/C, V: G/A/C, Y: C/T. The artificially added nucleotides were underlined.

**Table 2:** The nucleotide and amino acid sequence comparison of six ORFs of JYMaV Aksu isolates HZ, AKS-6 and AKS-15 with those of reported isolate SY.

ORF	Isolate	nt	nt%	aa	aa%
ORF1	SY	6930		2309	
	HZ	6930	93.3	2309	97.3
	AKS-6	6930	98.3	2309	99.3
	AKS-15	6930	98.6	2309	99.4
ORF2	SY	1986		661	
	HZ	1974	89.6	657	91.8
	AKS-6	1974	88.0	657	90.7
	AKS-15	1974	89.5	657	90.9
ORF3	SY	885		294	
	HZ	885	95.6	294	95.2
	AKS-6	885	95.9	294	95.2
	AKS-15	885	95.5	294	94.9
ORF4	SY	1125		374	
	HZ	1131	91.5	376	95.4
	AKS-6	1131	91.8	376	95.4
	AKS-15	1131	91.6	376	95.4
ORF5	SY	837		278	
	HZ	837	94.4	278	94.6
	AKS-6	837	92.7	278	92.4
	AKS-15	837	94.7	278	95.7
ORF6	SY	585		194	
	HZ	585	94.4	194	93.8
	AKS-6	585	94.5	194	93.8
	AKS-15	585	94.2	194	94.3

**Table 3:** Incidence of JYMaV in the jujube leaf samples collected from Aksu area in Xinjiang Uygur Autonomous region and Shanxi Province of China by RT-PCR using three primer sets.

Origin	Variety	Infected/Tested ^a^			
		NA	CLS		
			3-F/3-R	5-F/5-R	6-F/6-R
Xinjiang	Huizao	0/1	24/26	24/26	24/26
	Junzao	0/3	19/21	19/21	17/21
	Spinosa	0	10/12	11/12	10/12
	Jixinzao	0	1/1	1/1	1/1
	Dongzao	0	1/1	1/1	1/1
	unknown	0/10	0	0	0
Shanxi	Hupingzao	0/10	0	0	0
Total		0/24	55/61	56/61	53/61

^a^ CLS: chlorotic leaf spot; NA: no visible symptom. All asymptomatic samples were also tested by using three primer sets.

## Data Availability

The data presented in this study are available in article and supplementary materials.

## Supplementary Materials

The following are available online at https://www.mdpi.com/1999-4915/13/1/25/s1, Figure S1. Stem-loop structures formed by the conserved nucleotides at 5′ and 3′ termini of jujube yellow mottle-associated virus (JYMaV) RNAs. Figure S2. Multiple alignment of nucleotides at the 3′ un-translation region (3′ UTR) of RNA1 sequences of jujube yellow mottle-associated virus (JYMaV) amplified from Aksu jujube samples and the corresponding sequence of a reported isolate SY. The 21-bp repeat sequences were highlighted with red boxes. Each sequence was labeled with a sampling field ID followed with a location number and a clone number. Figure S3. Multiple alignments of nucleotides at the 5′ UTR of RNA3 sequences of jujube yellow mottle-associated virus (JYMaV) amplified from Aksu jujube samples and the corresponding sequence of a reported isolate SY. Each sequence was labeled with a sampling field ID followed with a location number and a clone number. Figure S4. Multiple alignments of nucleotides at the 5′ UTR of RNA4 sequences of jujube yellow mottle-associated virus (JYMaV) amplified from Aksu jujube samples and the corresponding sequence of a reported isolate SY. Each sequence was labeled with a sampling field ID followed with a location number and a clone number. Figure S5. Multiple alignments of nucleotides at the 5′ UTR of RNA5 sequences of jujube yellow mottle-associated virus (JYMaV) amplified from Aksu jujube samples and the corresponding sequence of a reported isolate SY. Each sequence was labeled with a sampling field ID followed with a location number and a clone number. Figure S6. Multiple alignments of nucleotides at the 5′ UTR of RNA6 sequences of jujube yellow mottle-associated virus (JYMaV) amplified from Aksu jujube samples and the corresponding sequence of a reported isolate SY. Each sequence was labeled with a sampling field ID followed with a location number and a clone number. Table S1. Primers designed for amplification of the ORF4 and ORF5 of jujube yellow mottle-associated virus (JYMaV) isolate HZ. Table S2. Contigs derived samples HZ by small RNA sequencing (sRNA-Seq) and matched proteins of raspberry leaf blotch virus (RLBV), a member of the genus *Emaravirus* as analyzed against database available in NCBI GenBank. Table S3. Contigs derived from samples AKS-6 and AKS-15 by RNA-Seq and identity with the genomic RNAs of jujube yellow mottle-associated virus (JYMaV) isolate HZ. Table S4. RT-PCR detection of jujube yellow mottle-associated virus (JYMaV) using the three pair of primers in jujube plants grown in Xinjiang Uygur Autonomous region of China and Shanxi Province.
