# Supplementary material for: Molecular Characteristics of Jujube Yellow Mottle-Associated Virus Infecting Jujube (Ziziphus jujuba Mill.) Grown at Aksu in Xinjiang of China
Source: Viruses. 2020 Dec 25;13(1):25. doi: 10.3390/v13010025 (PMC7823511; doi:10.3390/v13010025)
Supplement: Supplementary file 1 [file viruses-13-00025-s001.pdf]

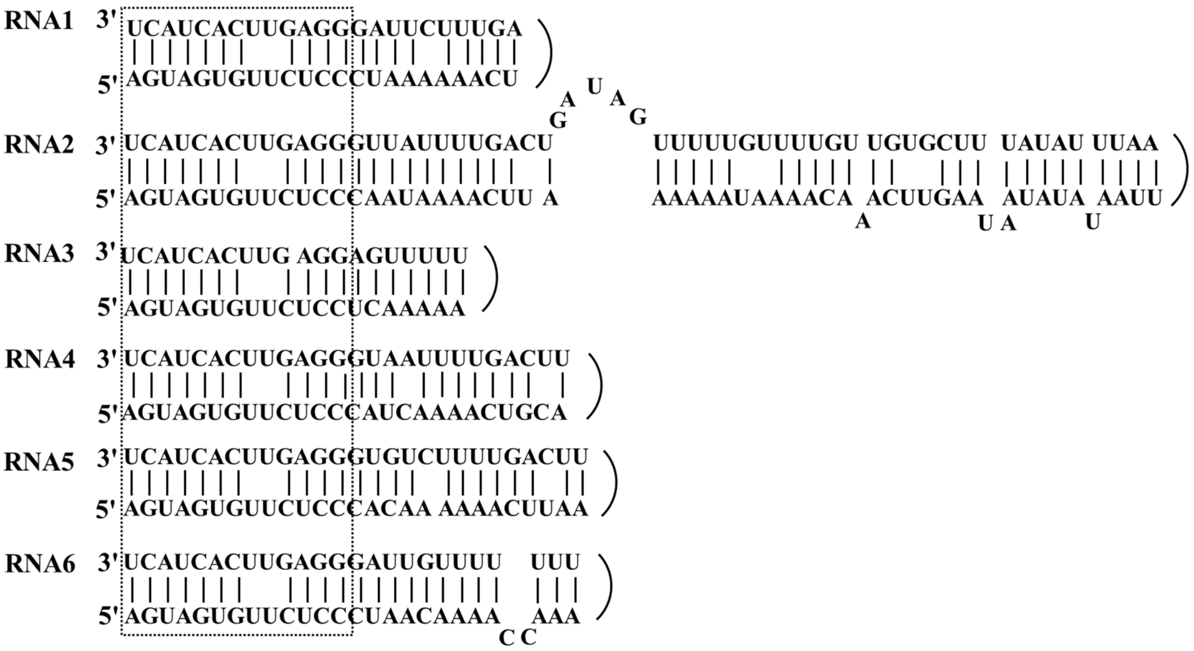

Figure S1. Stem-loop structures formed by the conserved nucleotides at 5′ and 3′ termini of jujube yellow mottle-associated virus (JYMaV) RNAs.

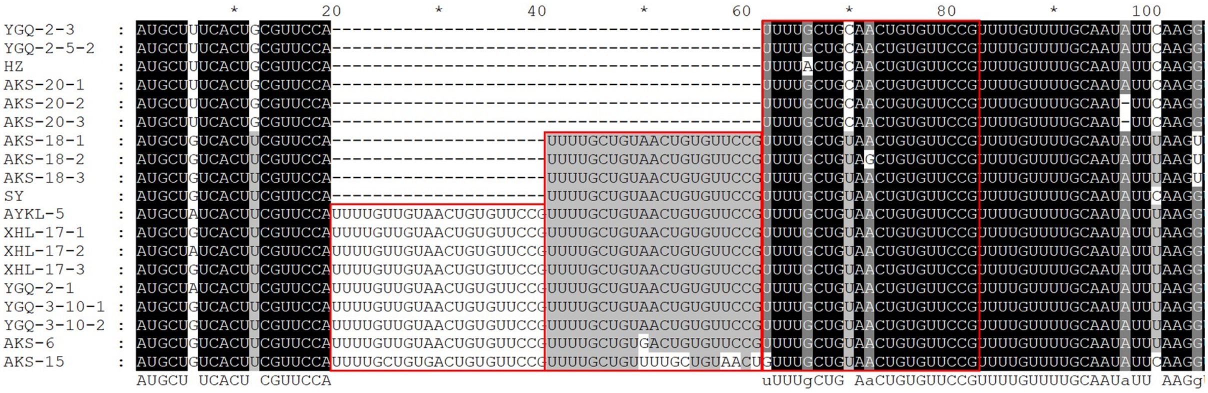

Figure S2. Multiple alignment of nucleotides at the 3′ un-translation region (3'UTR) of RNA1 sequences of jujube yellow mottle-associated virus (JYMaV) amplified from Aksu jujube samples and the corresponding sequence of a reported isolate SY. The 21-bp repeat sequences were highlighted with red boxes. Each sequence was labeled with a sampling field ID followed with a location number and a clone number.

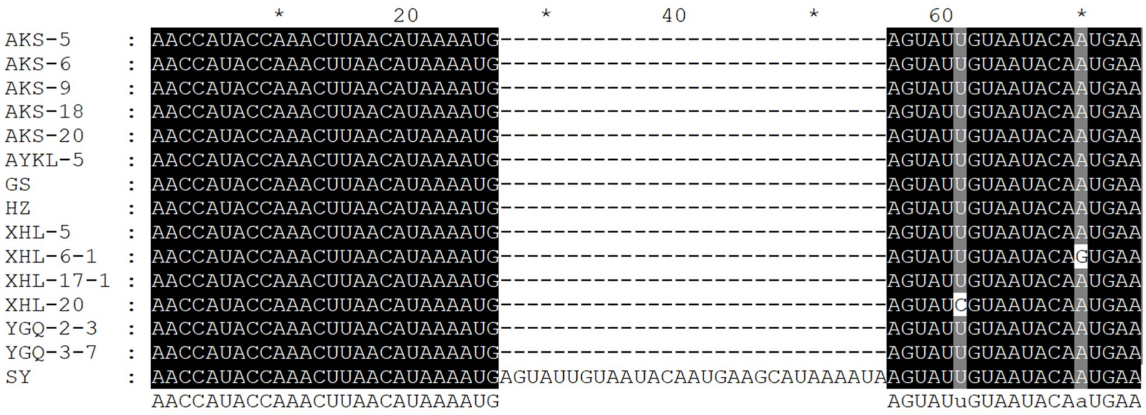

Figure S3. Multiple alignments of nucleotides at the 5' UTR of RNA3 sequences of jujube yellow mottle-associated virus (JYMaV) amplified from Aksu jujube samples and the corresponding sequence of a reported isolate SY. Each sequence was labeled with a sampling field ID followed with a location number and a clone number.

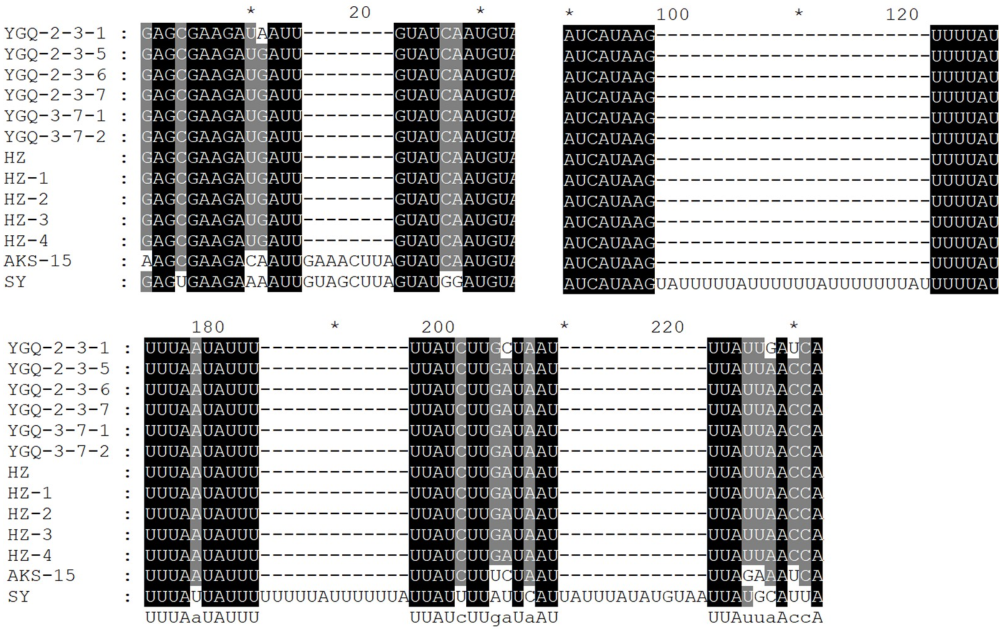

Figure S4. Multiple alignments of nucleotides at the 5' UTR of RNA4 sequences of jujube yellow mottle-associated virus (JYMaV) amplified from Aksu jujube samples and the corresponding sequence of a reported isolate SY. Each sequence was labeled with a sampling field ID followed with a location number and a clone number.

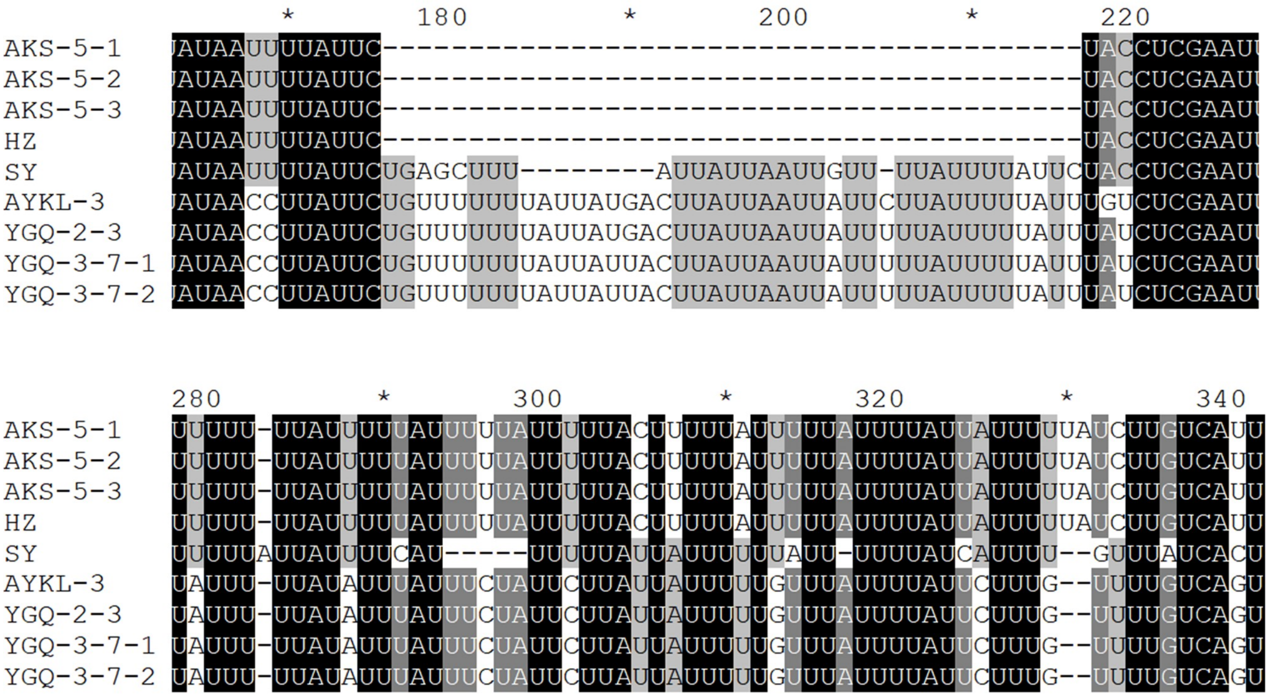

Figure S5. Multiple alignments of nucleotides at the 5' UTR of RNA5 sequences of jujube yellow mottle-associated virus (JYMaV) amplified from Aksu jujube samples and the corresponding sequence of a reported isolate SY. Each sequence was labeled with a sampling field ID followed with a location number and a clone number.

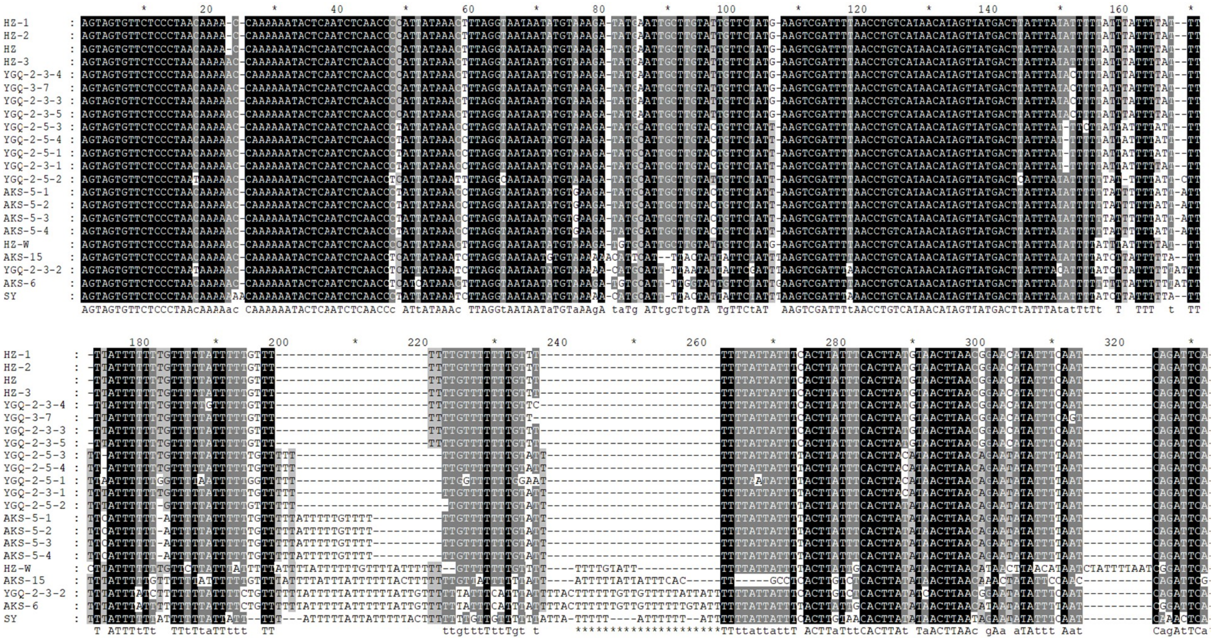

Figure S6. Multiple alignments of nucleotides at the 5' UTR of RNA6 sequences of jujube yellow mottle-associated virus (JYMaV) amplified from Aksu jujube samples and the corresponding sequence of a reported isolate SY. Each sequence was labeled with a sampling field ID followed with a location number and a clone number.

Table S1 Primers designed for amplification of the ORF4 and ORF5 of jujube yellow mottle-associated virus (JYMaV) isolate HZ

ORF	Primer	Sequence [a] (5′–3′)	Position (nt)	Product size (bp)
ORF4	P4-F	AAAAAGCAGGCTCCATGAAAATATTTCAAGTATATT	1317–1392	1155
	P4-R	AGAAAGCTGGGTCCAATGCTATCTTAGACATATC	265–285	
ORF5	P5-F	AAAAAGCAGGCTCCATGAAAGTTATCAAGGCTTAC	1131–1151	861
	P5-R	AGAAAGCTGGGTCATCATTATAATGTATGGAC	318–336	

a: The artificially added nucleotides used for recombination reactions were underlined.

Table S2. Contigs derived samples HZ by small RNA sequencing (sRNA-seq) and matched proteins of raspberry leaf blotch virus (RLBV), a member of the genus *Emaravirus* as analyzed against database available in NCBI GenBank

Matched protein	No. of contig	Length (bp)	aa identity（%）
P1	51	51-287	48.4-90.8
P2	12	56-180	51.6-70.8
P3	12	56-190	59.4-88.2
P4	13	55-350	46.8-91.3
P8	1	73	69.2

Table S3 Contigs derived from samples AKS-6 and AKS-15 by RNA-seq and identity with the genomic RNAs of jujube yellow mottle-associated virus (JYMaV) isolate HZ

Sample	RNA	Contig ID	No. of reads	Size (bp)	Position（nt）	Identity (%)
AKS-6	RNA1	contig_364	280980	7134	31–7160	92.2
	RNA2	contig_489	90265	2221	1–2221	95.9
	RNA3	contig_60	298358	1225	1–1224	98.3
	RNA4	contig_255	65443	1312	168–1483	91.6
	RNA5	contig_309	109523	1260	11–1274	88.9
	RNA6	contig_66	116627	991	1–991	90.1
AKS-15	RNA1	contig_177	370602	7151	13–7159	92.4
	RNA2	contig_70	93402	2221	1–2221	93.7
	RNA3	contig_107	601278	1228	1–1228	98.0
	RNA4	contig_184	86128	1488	2–1488	95.8
	RNA5	contig_914	79172	1239	2–1239	97.9
	RNA6	contig_305	99406	975	1–975	87.9

Table S4 RT-PCR detection of jujube yellow mottle-associated virus (JYMaV) using the three pair of primers in jujube plants grown in Xinjiang Uygur Autonomous region of China and Shanxi Province

Origin	Variety	Sample ID[a]	Tissue[b]	Symptom[c]	RT-PCR[d]		
					RNA3	RNA5	RNA6
Xinjiang	Huizao	AKS-1	L	CRS	+	+	+
		AKS-3	L	CRS	-	-	-
		AKS-5	L	B, CS	+	+	+
		AKS-9	L	M, MF	+	+	+
		HZ-1	L	CRS, MF, B	+	+	+
		XHL-17	L	CS, MF, M	+	+	+
		XHL-18	L	CS, MF, M	+	+	+
		XHL-19	L	CS, MF, M	+	+	+
		XHL-20	L	CS, MF, M	+	+	+
		YGQ3-1	L	CS, MF, M	+	+	+
		YGQ3-2	L	CS, MF, M	+	+	+
		YGQ3-3	L	CS, MF, M	+	+	+
		YGQ3-4	L	CS, MF, M	-	-	-
		YGQ3-5	L	CS, MF, M	+	+	+
		YGQ3-6	L	CS, MF, M	+	+	+
		YGQ3-7	L	CS, MF, M	+	+	+
		YGQ3-8	L	CS, MF, M	+	+	+
		YGQ3-9	L	CS, MF, M	+	+	+
		YGQ3-10	L	CS, MF, M	+	+	+
		YGQ3-11	L	CS, MF, M	+	+	+
		YGQ3-12	L	CS, MF, M	+	+	+
		YGQ3-13	L	CS, MF, M	+	+	+
		YGQ3-14	L	CS, MF, M	+	+	+
		YGQ3-15	L	CS, MF, M	+	+	+
		YGQ2-7	L	CS, MF, M	+	+	+
		YGQ2-8	L	CS, MF, M	+	+	+
		KEL-1	L	N	-	-	-
		AKS-8	L	B, CS	+	+	+
		AKS-10	L	N	-	-	-
		AKS-11	L	N	-	-	-
		AKS-12	L	CS, B	+	-	-
		AKS-14	L	CS, B	+	+	+
		AKS-15	L	CS, MF	+	+	+
		AKS-16	L	CS, B	+	+	+
		AKS-17	L	CS, B	+	+	+
		AKS-18	L	M, B	+	+	+

		AKS-19	L	CS, B	+	+	+
		AKS-20	L	M, B	+	+	+
		XHL-1	L	CS, MF, M	+	+	+
		XHL-2	L	CS, MF, M	+	+	+
		XHL-3	L	CS, MF, M	-	+	+
		XHL-4	L	CS, MF, M	+	+	+
		XHL-5	L	CS, MF, M	+	+	-
		XHL-10	L	CS, MF, M	+	+	+
		YGQ2-1	L	CS, MF, M	+	+	-
		YGQ2-2	L	CS, MF, M	-	-	-
		YGQ2-3	L	CS, MF, M	+	+	+
		YGQ2-4	L	CS, MF, M	+	+	+
		YGQ2-5	L	CS, MF, M	+	+	+
		YGQ2-6	L	CS, MF, M	+	+	+
		KEL-2	L	N	-	-	-
	Z. jujube	AKS-2	L	CRS	+	+	+
	var. spinosa	AKS-4	L	CRS	+	+	+
		AKS-6	L	CRS	+	+	+
		XHL-6	L	CS, MF, M	+	+	+
		XHL-7	L	CS, MF, M	+	+	+
		XHL-8	L	CS, MF, M	-	-	-
		XHL-9	L	CS, MF, M	-	+	-
		AYKL-1	L	CS, MF, M	+	+	+
		AYKL-2	L	CS, MF, M	+	+	+
		AYKL-3	L	CS, MF, M	+	+	+
		AYKL-4	L	CS, MF, M	+	+	+
		AYKL-5	L	CS, MF, M	+	+	+
	Jixinzao	AKS-7	L	B, MF, CS	+	+	+
	Unknow	WS-1	L	N	-	-	-
		WS-2	L	N	-	-	-
		WS-3	L	N	-	-	-
		WS-4	L	N	-	-	-
		WS-5	L	N	-	-	-
		WS-6	L	N	-	-	-
		WS-7	L	N	-	-	-
		WS-8	L	N	-	-	-
		WS-9	L	N	-	-	-
		WS-10	L	N	-	-	-
	Dongzao	AKS-13	L	CRS	+	+	+
Shanxi	Hupingzao	SX-1	L	N	-	-	-
		SX-2	L	N	-	-	-
		SX-3	L	N	-	-	-

	SX-4	L	N	-	-	-
	SX-5	L	N	-	-	-
	SX-6	L	N	-	-	-
	SX-7	L	N	-	-	-
	SX-8	L	N	-	-	-
	SX-9	L	N	-	-	-
	SX-10	L	N	-	-	-
Total				55	56	53

a: samples were collected from five different locations in Xinjiang Uygur Autonomous region of China as indicated by the first letters (AKS, XHL, YGQ3, YGQ2, and AYKL) in the sample names.

b: L, leaf

d: M, mosaic; B, blotch; MF, malformation; CS, chlorotic spot; CRS, chlorotic ringspot; N, symptom unknown.

d: +, positive; -, negative; N, not tested.
